# The Mission of Long Non-Coding RNAs in Human Adult Renal Stem/Progenitor Cells and Renal Diseases

**DOI:** 10.3390/cells12081115

**Published:** 2023-04-08

**Authors:** Francesca Giannuzzi, Silvia Maiullari, Loreto Gesualdo, Fabio Sallustio

**Affiliations:** 1 Department of Interdisciplinary Medicine (DIM), University of Bari Aldo Moro, 70124 Bari, Italy; 2 Department of Precision and Regenerative Medicine and Ionian Area (DiMePRe-J), University of Bari Aldo Moro, 70124 Bari, Italy; 3 MIRROR—Medical Institute for Regeneration, Repairing and Organ Replacement, Interdepartmental Center, University of Bari Aldo Moro, 70124 Bari, Italy

**Keywords:** long non-coding RNAs, renal disease, stem cell biology, adult renal progenitor cells, lncRNA HOTAIR

## Abstract

Long non-coding RNAs (lncRNAs) are a large, heterogeneous class of transcripts and key regulators of gene expression at both the transcriptional and post-transcriptional levels in different cellular contexts and biological processes. Understanding the potential mechanisms of action of lncRNAs and their role in disease onset and development may open up new possibilities for therapeutic approaches in the future. LncRNAs also play an important role in renal pathogenesis. However, little is known about lncRNAs that are expressed in the healthy kidney and that are involved in renal cell homeostasis and development, and even less is known about lncRNAs involved in human adult renal stem/progenitor cells (ARPC) homeostasis. Here we give a thorough overview of the biogenesis, degradation, and functions of lncRNAs and highlight our current understanding of their functional roles in kidney diseases. We also discuss how lncRNAs regulate stem cell biology, focusing finally on their role in human adult renal stem/progenitor cells, in which the lncRNA HOTAIR prevents them from becoming senescent and supports these cells to secrete high quantities of α-Klotho, an anti-aging protein capable of influencing the surrounding tissues and therefore modulating the renal aging.

## 1. Introduction

Deep sequencing methods have shown that many non-coding RNAs (ncRNAs) exist and that the majority of the eukaryotic genome is transcribed. Since RNA molecules are perfectly suited to selectively recognize other RNAs and DNA by complementary base pairing, ncRNAs are numerous and highly adapted in their roles in contemporary organisms. Thousands of ncRNAs with little or no protein-coding ability are part of this intricate network of transcripts. They serve as post-transcriptional regulatory molecules that interact with particular mRNAs to control the production of proteins. The ncRNAs can also interact with proteins or other nucleic acids, affecting secondary and tertiary structures that are essential for their proper functioning. These most recent discoveries go beyond Francis Crick’s 1957 DNA-RNA-protein basic dogma [1]. The lncRNAs are a large, heterogeneous class of transcripts, longer than 200 nucleotides, and key regulators of gene expression at both the transcriptional and the post-transcriptional level in different cellular contexts and biological processes [2,3]. This group of transcripts shares features with coding transcripts (mRNAs). Contrary to their smaller counterparts, lncRNAs exhibit cell type-specific expression, localization to subcellular compartments, and are significantly less conserved. It is interesting to note that the majority of lncRNAs are linked to human disorders, much like miRNAs. In terms of physiological regulation, lncRNAs have been demonstrated to affect all aspects of gene regulation, including but not limited to promoter activity, epigenetics, the effectiveness of translation and transcription, and intracellular trafficking [4]. The kidney expresses tens of thousands of lncRNA sequences, which are frequently conserved with coding genes, just like other tissues. Numerous studies have demonstrated that lncRNAs express at various levels and are implicated in all phases of renal disorders. Targeting lncRNAs may be a precise treatment approach for developing kidney illnesses. LncRNAs have been identified as diagnostic biomarkers [5,6,7]. LncRNAs may be present as persistent biomarkers in serum and urine [8,9,10]. The key to their utility as biomarkers is that they exhibit variable levels of expression in various diseases and are linked to pathways, targets, and events involved in the pathophysiology of kidney disease.

Here we give a thorough overview of the biogenesis, degradation, and functions of lncRNAs and highlight our current understanding of their functional roles in kidney diseases, such as chronic kidney disease (CKD), membranous nephropathy (MN), immunoglobulin A nephropathy (IgAN), lupus nephritis (LN), diabetic nephropathy (DN), and acute kidney injury (AKI). We also discuss how lncRNAs regulate stem cell biology, focusing finally on their role in human adult renal stem/progenitor cells.

## 2. Biogenesis, Structure, and Functions of Long Non-Coding RNAs (LncRNAs)

The biogenesis of lncRNAs is cell-type and stage-specific [11], and different classes of lncRNA transcripts can be identified depending on the transcription locus, i.e., promoter upstream regions (PROMPTs), enhancers (eRNAs), intervening/intergenic regions (lincRNAs), and the opposite strand of protein-coding genes (NATs), respectively [12]. Briefly, PROMPTs are antisense transcripts of 0.5–2.5 kb, upstream of the active transcription start sites (TSSs) of mammalian protein-coding genes [13]. The PROMPTs function is still unknown, but their expression levels are strongly affected by stressful conditions and are largely retained in the nucleus [14]. The eRNAs are bidirectional transcripts generated from enhancer sequences by Pol II and are usually less than 2000 nucleotides in length [14]. This group of lncRNAs has enhancer-like functions in gene regulatory networks by controlling promoter and enhancer interactions [15,16]. LincRNAs are the most-studied lncRNAs, contain multiple exons, and have typical mRNA-like features. They are produced by Pol II activity on intergenic regions between two genes [17], and their structural similarity with the mRNAs suggests that the function is also analogous.

**Figure 1:**
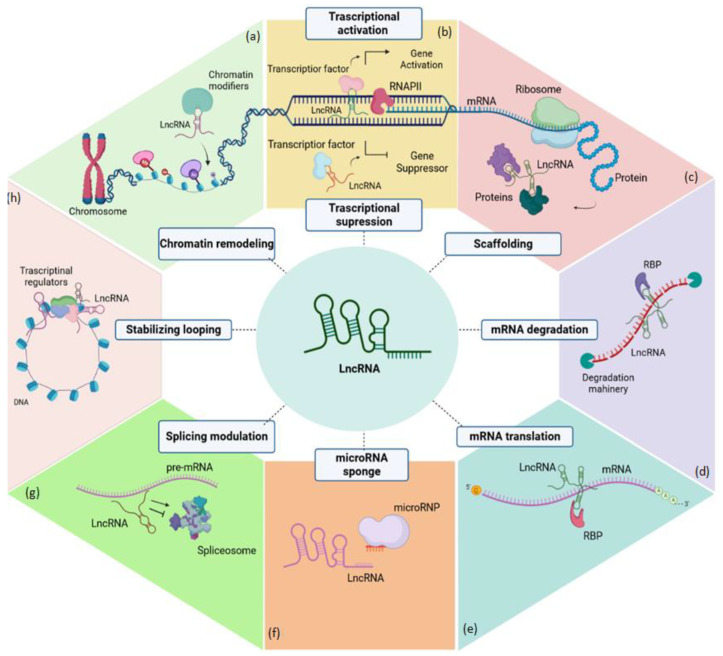
Classification of lncRNA functions. LncRNAs act as regulators of gene expression: (**a**,**h**) they can influence chromatin architecture by interacting with different protein components of the remodeling complex and modifying chromatin organizational patterns. (**b**) They activate the transcription of certain genes by driving transcription factors to their promoters and by targeting transcriptional modulators such as RNA polymerase (RNAP) II; (**c**) however, they are also capable of suppressing transcription by sequestering transcription factors and keeping them away from their promoters. LncRNAs also control various aspects of post-transcriptional mRNA processing, including: (**d**) they perform scaffolding roles by providing docking sites for proteins that function together in the same biological pathway; (**e**) through binding to protein interactors, including classical RNA-binding proteins (RBPs), they are able to modulate mRNA functioning by also subjecting them to degradative pathways; (**f**) they act like “sponges” by base pairing with their complementary miRNAs and reducing their effects; (**g**) they alter their splicing patterns.

The functions of lncRNAs in the cellular context are closely associated with their subcellular localization and compartmentalization. In fact, lncRNAs actively influence the transcription process and chromatin organization when they carry out their bioactivity in the nucleus, while they can affect the stability and post-translational modifications of the mRNAs in the cytoplasm (Figure 1). In detail, the lncRNAs are largely retained in the nucleus mainly due to the presence of elements in cis and short C-rich sequences, primate-specific short interspersed nuclear elements (SINEs), which interact with nuclear proteins [18,19]. In the nucleus, lncRNAs can modulate inter- and intrachromosomal interactions [20,21]. Moreover, some lncRNAs are able to prevent or promote the recruitment of chromatin modifiers by influencing chromatin remodeling either in cis (near their transcription sites) or in trans (at sites distant from their transcription sites), mediating histone methylation or histone ubiquination [22], and facilitating the recruitment of chromatin regulatory complexes [23,24,25].

Furthermore, the lncRNAs can also directly regulate gene transcription using two different mechanisms. The first known molecular mechanism is the formation of R-loop structures, which are triple-stranded nucleic acid structures with RNA hybridized. The second mechanism of gene expression control is interference with Pol II transcription machinery (Figure 1) [26,27,28,29].

As previously said, lncRNAs perform important functions in the cell cytoplasm as well. In particular, lncRNAs in the cytoplasm can stabilize mRNAs, influence translation, and modulate post-translational modification (Figure 1). In particular, lncRNAs can influence mRNA turnover through several mechanisms. The first of these is the ability to recruit proteins for mRNA degradation. The mRNAs regulated by this mechanism contain *Alu* elements within their 3′-UTRs that are complementary to *Alu* present in the lncRNAs sequence. The complementarity leads to the formation of structures, double-stranded RNAs (dsRNAs), that are recognized by Staufen 1 (STAU1), leading to mRNA decay [30,31]. The second mechanism is constituted by the recalling of RNA-binding proteins (RBPs) involved in mRNA degradation (Figure 1). One of these regulatory lncRNAs is noncoding RNA activated by DNA damage (NORAD), which stimulates mRNA deadenylation and decapping, resulting in accelerated turnover [32,33]. Finally, lncRNAs can function as sponges for miRNAs, competing for miRNA binding and decreasing their availability for molecular targets [32,33,34] (Figure 1). This class of lncRNAs is identified as competitive endogenous RNAs (ceRNAs), and the molecular mechanisms are still not completely elucidated. It is also important to point out that the same lncRNA can have more gene targets [35], but also that different lncRNAs could have the same gene target. Recent studies devised a “chromatin-RNA reverse transcription in situ sequencing” (CRIST-seq) approach to profile the lncRNA interaction network in gene regulatory elements, uncovering a true lncRNA interaction network specific for the pluripotency in the Sox2 and Pou5f1 promoters [36]. The regulatory role of differentially expressed HOX-interacting lncRNAs in various biological processes and cancer hallmark events has been demonstrated by building interaction networks of lncRNA [37].

Although the functions of most lncRNAs are largely unknown, the impressive advances in a short time in the knowledge of lncRNAs underline the enormous potential of these RNAs as potential non-protein biomarkers. In this regard, cell-free nucleic acids or circulating nucleic acids (CNAs) have recently been proposed as a new class of potential biomarkers that could improve the diagnosis, especially in the oncologic field [38]. The stability of lncRNAs in the bloodstream, thanks to the presence of extensive secondary structures and transport by protective exosomes [39,40], and the ease of detection designate these RNAs as very reliable potential biomarkers. Numerous studies have reported the imbalance of lncRNA expression in cancer [41,42,43]. HULC, H19, HOTAIR, and GACAT2 (for “gastric cancer-associated transcript 2”) were found to be significantly increased in the plasma of gastric cancer (GC) patients compared to healthy individuals [44,45,46]. Moreover, a high level of HOTAIR has also been detected in samples from colorectal cancer patients [47].

Given the important regulatory role of lncRNAs at the cellular level, thinking of them as passive biomolecules for the detection of diseases only would be extremely limiting. In fact, they may also represent very innovative and effective therapeutic approaches or targets for several types of diseases, starting with cancer, in which they have been extensively studied [48,49,50]. However, many studies will still be needed to elucidate whether targeting circulating lncRNAs will be sufficient for tumor treatment. Indeed, one limit can be represented by the nucleic acids’ inability to cross the cell membrane due to their negative charge due to the phosphate groups. This certainly could limit the penetrability of lncRNAs to cells and tissues. At the same time, the transport by lipid-based extracellular vesicles could guarantee greater efficiency of the treatment. LncRNAs represent 3.36% of the RNA content of the exosomes, which are known carriers of biomolecules that cells exploit for paracrine, endocrine, and autocrine communication throughout the whole body [51]. The complex exosome-based delivery system could therefore be used to provide specific oncosuppressor lncRNAs, normally downregulated in cancer, ensuring their spread and diffusion in tumor-affected tissues. However, other lncRNAs may be transported from the systemic circulation in complexes with other proteins (Argonaute-Ago or nucleophosmin 1-NPM1) [52] or even in unhybridized forms. These classes of lncRNAs could be targeted with interference [53].

All these future application perspectives of lncRNAs could be transferred from the oncological field to other clinical fields as well, given the involvement of lncRNAs in numerous other biological processes, both physiological and pathological.

## 3. The Role of LncRNA in Kidney Disease

Altered expression of lncRNAs has been increasingly closely related to the onset and development of many diseases due to their role in gene regulation processes at the transcriptional, post-transcriptional, translational, post-translational, and epigenetic levels. Therefore, increasing attention is being paid to their role as diagnostic and prognostic biomarkers and therapeutic targets in several human diseases. Regarding kidney diseases, there are numerous studies that have analyzed and demonstrated the role of lncRNAs mainly in DN and AKI, and to a lesser extent in CKD, focal segmental glomerulosclerosis (FSGs), and IgAN (Table 1).

### 3.1. Diabetic Nephropathy

Diabetic nephropathy (DN) is a chronic kidney disease that results from diabetes mellitus and is characterized by albuminuria, a decline in the glomerular filtration rate, and arterial hypertension. If left untreated, it can progress to end-stage renal disease (ESRD) [54,55]. Plasmacytoma variant translocation 1 (PVT1) was one of the first lncRNAs associated with DN. Through genome-wide SNP genotyping analyses, PVT1 was indicated as a susceptibility locus for the onset of DN and the development of ESRD [56]. High glucose content upregulated the expression of PVT1 in mesangial cells, causing increased expression of proteins that composed the extracellular matrix (ECM). Conversely, silencing of PVT1 led to a significant decrease in mRNA and major ECM proteins, such as fibronectin and collagen type IV alpha 1, as well as their transcriptional regulators, such as transforming growth factor beta 1 (TGFB1) and plasminogen activator inhibitor (PAI-1). These findings suggest that PVT1 may mediate the development and progression of DN through mechanisms involving ECM protein accumulation [57,58]. One of the mechanisms underlying the pathogenesis of DN is the disruption of mitochondrial homeostasis. Long and et al. linked the modulation of mitochondrial metabolism to the lncRNA TUG1 [59]. Specifically, they observed that TUG1 was linked to mitochondrial bioenergetics by recruitment of PGC-1α (Peroxisome proliferator-activated receptor-γ Coactivator-1 α) to its promoter. Transgenic mice that overexpressed TUG1 in podocytes were protected from diabetes-induced CKD, while glomerular TUG1 levels were reduced in both mice and renal biopsies from diabetic patients. Moreover, specific overexpression of TUG1 in podocytes from this mouse model improved the glomerular phenotype with regard to both albuminuria and histological changes. This suggests that this lncRNA may be a possible therapeutic target to treat kidney disease and/or diabetes [59,60]. Yang et al. observed that 45 and 813 lncRNAs were up- and downregulated, respectively, in the serum of DN patients compared with diabetic patients [61]. Among them, lncRNA-ARAP1-AS2 and lncRNA-ARAP1-AS1 are the ones involved in the pathogenesis of DN. LncRNA-ARAP1-AS2 gradually increases during the progression of diabetes and diabetic nephropathy, while lncRNA-ARAP1-AS1 gradually decreases. Both enhance the mRNA expression of ARAP1, a member of the renin-angiotensin system (RAS) [61,62]. Several studies indicate a functionally important involvement of NEAT1 lncRNA in diabetic nephropathy. Increased expression of NEAT1 contributes to proliferation and fibrosis in the progression of DN through activation of Akt/mTOR signaling, whereas expression of TGF-β1, FN, and COL-IV is repressed by NEAT1 in vitro [63].

### 3.2. Acute Kidney Injury

Acute kidney injury is a complex renal disorder characterized by an abrupt decline in renal function. The main triggers of AKI are sepsis, nephrotoxic insults, and ischemia-reperfusion. Despite considerable progress, the AKI pathophysiological mechanisms have not been fully explored. Numerous pieces of evidence have accumulated showing that non-coding RNAs are involved in the pathophysiology of AKI and the regulation of numerous genes, showing significant potential for the development of diagnostic and therapeutic strategies. Most studies conducted to examine lncRNAs in AKI have been performed in vivo in mice or rats by induction of urine-derived sepsis [64,65], ischemia-reperfusion injury (IRI) [66,67,68], and lipopolysaccharide (LPS)-stimulated inflammation [69,70], while the human tubular epithelial cell line HK-2, treated with LPS [64,65], or grown under hypoxic conditions [67,68,71], has been used in vitro (Table 1).

In a microarray study, it was observed that 5361 lncRNAs were upregulated and 5928 were downregulated in patients with septicemia-induced AKI. Among the various lncRNAs studied, MALAT1 and TUG1 also occur. MALAT1 expression is increased in the serum of patients with sepsis, in the kidney tissue of experimental animals, and in LPS-treated kidney cells. MALAT1 promotes renal damage by activating nuclear factor-κB (NF-κB). Accordingly, silencing of MALAT1 showed a significant renal protective effect [69]. In contrast to the above-mentioned lncRNA MALAT1, overexpression of TUG1 showed a protective effect in LPS-treated HK-2 cells by modulating the NF-kB gene [72] and through the interaction with the nuclear factor erythroid 2-related factor 2 (Nrf2) transcription factor [73]. In addition, HOXA-AS2 lncRNA showed protection in sepsis-caused AKI by hindering Wnt/β-catenin and NF-κB pathways [74]. In addition, overexpression of lncRNA6406 attenuates cellular inflammation, oxidative stress, and apoptosis through modulation of miR-687/PTEN signaling [75,76]. XLOC-032768 and LRNA9884 lncRNAs were studied in AKI induced by nephrotoxic agents in vivo and in vitro. Zhou et al. demonstrated that overexpression of lncRNA XLOC-032768 reduced apoptosis and TNF-mediated inflammation in mice and cells exposed to cisplatin [77], while Zhang et al. showed that LRNA9884 was markedly upregulated in the nucleus of the renal tubular epithelium in mice with AKI and promoted inflammatory cytokine production via NF-κB [78].

Another important cause of AKI is ischemia/reperfusion (IR). XIST, NEAT1, MALAT1, and H19 lncRNAs have been found upregulated in human biopsies of AKI, in experimental models of IR, and in cultured hypoxic endothelial and tubular cells [79,80,81,82] (Table 1). Increased lncRNA XIST (X inactive specific transcript) in IR-damaged kidneys and renal cells induces apoptosis and inflammation [79]. NEAT1 induces apoptosis of renal tubular epithelial cells through downregulation of miR-27a-3p, identifying this miRNA as a target of NEAT1 [82]. Overexpression of H19 lncRNA improved renal function and angiogenesis and decreased inflammation and apoptosis through upregulation of miR-30a-5p [80]. The upregulation of MALAT1 was activated by hypoxia-inducible factor 1-α (HIF-1α) and negatively regulated the expression of IL-6, TNF-α, and NF-kB [81]. The lncRNA PRINS is involved in the AKI process by regulating the production of RANTES, a major inflammatory mediator of AKI following IR injury. Increased levels of RANTES in renal tubular cells further aggravated renal injury through recruitment of inflammatory cells and led to loss of renal function after IR injury [83].

### 3.3. Chronic Kidney Disease

CKD is a disease characterized by hypoperfusion-induced tubular ischemia, interstitial fibrosis, and impaired renal function [84,85]. Additionally, in this case, several lncRNAs that can regulate the expression of some CKD-related genes and proteins, such as collagen, smooth muscle α-actinin, and fibronectin, have been identified (Table 1). For example, the lncRNAs TCONS_00088786 [86] and TCONS_01496394 [87], which are regulated by TGF-β stimulation, can influence the expression of some fibrosis-related genes through a feedback loop. Similarly, lncRNA Erbb4-immunoreactivity (Erbb4-IR) is induced by TGF-β1 and highly upregulated in fibrotic kidneys. The silencing of Erbb4-IR blocks TGF-β1–induced collagen I and α–smooth muscle actin expression in vitro [88] and up-regulates Smad7 in the kidneys, thereby attenuating TGF-β1/Smad3-induced renal fibrosis in vivo and in vitro [89,90].

PVT1 lncRNA is mainly regulated by the miR-181a-5p/TGF-βR1 signaling pathway. The expression of PVT1 lncRNA is significantly upregulated in renal fibrosis. Knockdown of lncRNA PVT1 inhibited the progression of renal fibrosis via regulation of TGF-β signaling, downregulation of the expression of α-SMA, upregulation of the expression of E-cadherin, and via miR-181a-5p [91]. H19 lncRNA, together with miR-17 and fibronectin, forms a regulatory network involved in renal fibrosis [92]. Its expression has been significantly correlated with oxidative stress and inflammatory markers such as tumor necrosis factor-α (TNF-α) and interleukin (IL)-6 in patients with CKD [93,94].

Other lncRNAs may influence the expression of some inflammatory factors and are associated with the production of and defense against reactive oxygen species (ROS). The lncRNA XIST (X inactive specific transcript) attenuates renal inflammation, and ROS production induces oxidative damage in renal calcinosis [95]. In addition, lncRNAs can drive renal fibrosis by regulating several biological processes, such as apoptosis, cell proliferation, autophagy, and epithelial-mesenchymal transition (EMT). One example is LINC00667 lncRNA, which reduces the proliferation and invasion of CKD cells while increasing the rate of apoptosis [96,97]. LncRNA 74.1 was significantly downregulated in clinical samples of renal fibrosis and promoted ROS defense by activating prosurvival autophagy, then reducing ECM-bound proteins fibronectin and collagen I involved in renal fibrosis [98].

LncRNAs are also involved in another important mechanism of pathological damage in CKD called pyroptosis. This process is a particular type of programmed cell death that includes some features of apoptosis and necrosis. The lncRNAs MALAT1 (metastasis-associated lung adenocarcinoma transcript 1) promote pyroptosis through downregulation of miR-23c, while GAS5 (growth arrest-specific 5) has anti-pyroptotic properties [79,99].

### 3.4. Glomerulonephritis

LncRNAs have been involved in different kinds of glomerulonephritis (Table 1). Focal segmental glomerulosclerosis is a common kidney disease resulting from the dysfunction and apoptosis of podocytes in the glomerulus of the kidney. Compared with DN, little is known about the contribution of lncRNAs to this glomerular disease. One of the lncRNAs associated with FSGS is lncRNA LOC105375913 [100], whose increased expression promotes snail overexpression and tubulointerstitial fibrosis. Upregulation of lncRNA LOC105374325, related to activation of the P38/C/EBPβ pathway in podocytes of individuals with FSGD, increases the level of Bax and Bak genes and causes cell apoptosis [101].

IgA nephropathy is one of the most common primary glomerulonephritis and is characterized by immune complexes (IC) formed mainly by IgA that are deposited in the mesangial area of the glomerulus, causing glomerular inflammation and further renal damage [102]. About 217 lncRNAs differentially expressed in peripheral blood monocyte cells (PBMCs) have been suggested as potential factors involved in the pathophysiology of IgA nephropathy [103]. Among them, HOTAIR has been the most important lncRNA in the regulation of differentially expressed genes/miRNAs in IgA nephropathy [103]. In another study, the lncRNA-G21551 was observed to be significantly down-regulated in IgAN patients and could play an important role in the pathogenesis of IgAN by regulating the expression of FCGR3B, a gene that encodes for the low affinity receptor (FcgR3B receptor) of the Fc segment of immunoglobulin G (IgG) [104]. Moreover, the lncRNA PTTG3P levels have been found higher in IgAN samples than in healthy subjects. Overexpression of PTTG3P induced B-cell growth, increased the expression of cyclin D1 and Ki-67 genes, and induced the production of IL-1β and IL-8, which play key roles in the onset and development of IgAN [105]. Some lncRNAs may be used as disease biomarkers. The expression of the lncRNA MYEF2-1.1 was 85-fold lower in IgAN patients than healthy controls, while that of ALOX15P1-ncNR045985 was 5.15-fold higher [106]. Wen et al. have observed a significant increase in intercellular adhesion molecule-1 (ICAM-1)-related lncRNA (ICR) levels in kidney tissue from patients with IgAN. This lncRNA is involved in the renal fibrotic processes; indeed, its inhibition attenuated the fibrotic changes in TGF-β1-induced renal proximal tubular cells through reduction of phosphorylation and consequent inhibition of the Akt/mTOR signaling pathway [107].

lncRNA XIST has been linked to membranous nephropathy [108,109], a kidney-specific autoimmune disease [110]. Its upregulation in a mouse model of MN and in human samples [109] has been associated with a proapoptotic effect on podocytes through upregulation of Toll-like receptor 4 and negative regulation of miR-217 [108]. Upregulation of NEAT1, on the other hand, promotes MN development by inhibiting the anti-apoptotic activity mediated by Noxa (a Bcl-2 homolog 3 protein) to induce apoptosis [111]. Finally, the lncRNA RP11-2B6.2 was found to be increased in the renal tissue of patients with lupus nephritis compared with healthy controls. Overexpression of RP11-2B6.2 led to inhibition of SOCS1, resulting in hyperactivation of the IFN-I signaling pathway in renal cells [112]. Among the many pathogenic signaling pathways identified in LN, hyperactivation of the IFN-I response is closely associated with disease progression and prognosis [113].

As noted so far, numerous studies have been conducted and knowledge gained on lncRNAs in the renal field in recent years, not only for their involvement in the pathophysiology of renal diseases but also for their potential as diagnostic and prognostic biomarkers and therapeutic targets. In addition, it is noteworthy that by analyzing lncRNA studies performed on renal diseases (Table 1), we can identify six lncRNAs involved in more than one type of renal disease, in particular PVT1, TUG1, NEAT1, MALAT1, XIST, and H19. Among these, PVT1 and XIST have a common mechanism on multiple kidney diseases, the first increasing the expression of extracellular matrix proteins through the TGFB and the second inducing apoptosis. The other four lncRNAs may have different mechanisms for each disease. Instead, this may be due to the type of study performed or the choice of the authors to study only a specific mechanism. Moreover, several lncRNAs have been found to be expressed only in one kind of renal disease. Additionally, in this case, it may depend on the type of study and on the experimental setting performed. However, despite considerable progress, there are still many unclear mechanisms and countless puzzles to be solved before study results can be promoted to the clinical level.

HK-2: Human Kidney 2; DN: Diabetic Nephropathy; TECs: Tubular Epithelial Cells; CKD: Chronic Kidney Disease; ECs: Endothelial Cells; AKI: Acute Kidney Injury; I/R: Ischemia/Reperfusion; PBMCs: Peripheral Blood Monocytes cells; IgAN: IgA Nephropathy; EMT: Epithelial-Mesenchymal Transition; PTEC: Proximal Tubular Epithelial Cells; FSGS: Focal Segmental Glomerulosclerosis; MN: Membranous Nephropathy; LN: Lupus Nephritis.

**Table 1:** Long non-coding RNAs in renal disease.

lncRNA	Tissue/Cells	Disease	Mechanism
ARAP1-AS1 and ARAP1-AS2	HK-2	DN	Both enhance the mRNA expression of ARAP1, a member of the renin-angiotensin system [62,63]
ENST-00000453774.1	HK-2	CKD	Reduces ECM-bound proteins fibronectin and collagen I [99]
Erbb4 –Immunoreactivity	TECs and elongated, fibroblast-like cells	CKD	Regulates the expression of collagen I, α–smooth muscle actin, and Smad7 [89]
GAS5	Kidney cells	CKD	Has anti-pyroptotic properties [100]
H19	ECs, TECs	AKI due to renal I/R injury	Upregulates miR-30a-5p [81]
	HK-2	CKD	Affects TNF-α and IL-6 expression [93,95]
HOTAIR	PBMCs	IgAN	Possible involvement in the NGF signaling pathway and Toll-like receptor pathways [104]
HOXA-AS2	HK-2	AKI	Hinders the Wnt/β-catenin and NF-κB pathways [75]
ICR	Renal Proximal Tubular Cells	IgAN	Is involved in the Akt/mTOR signaling pathway [108]
LINC00667	Renal Tubular Epithelial Cell	CKD	Regulates apoptosis, cell proliferation, autophagy, and EMT [97]
lncRNA 9884	HK-2	AKI induced by nephrotoxic agents	Promotes the production of inflammatory cytokines via NF-κB [79]
lncRN A6406	PTEC	AKI	Modulates miR-687/PTEN signaling [76,77]
lncRNA G21551	Exosomes	IgAN	Regulates the expression of FCGR3B [105]
lncRNA PTTG3P	Peripheral B cells	IgAN	Promotes B-cell growth, IL-1β, and IL-8 production by regulating miR-383 [106]
LOC105375913	Renal tubular cells	FSGS	Increases the level of snail and tubulointerstitial fibrosis [101]
LOC105374325	Podocytes	FSGS	Increases the level of Bax and Bak genes and causes cell apoptosis [102]
MALAT1	HK-2	AKI	Activates NF-κB [70]
	ECs, TECs	AKI due to renal I/R injury	Negatively regulates the expression of IL-6, TNF-α, and NF-kB [82]
	Kidney cells	CKD	Promotes pyroptosis by downregulating miR-23c [100]
NEAT1	Mesangial cells	DN	Activates Akt/mTOR signaling, and represses TGF-β1, FN, and COL-IV expression [64]
	ECs, TECs	AKI due to renal I/R injury	Downregulates miR-27a-3p [83]
	Renal Tubular Epithelial Cell	MN	Inhibits Noxa-mediated anti-apoptotic activity [112]
PRINS	Renal tubular cells	AKI due to renal I/R injury	Regulates the production of RANTES [84]
PVT1	Mesangial cells	DN	Increased expression of extracellular matrix proteins [57,58]
	HK-2	CKD	Is involved in the TGF-β signaling pathway [92]
RP11-2B6.2	Renal cells	LN	Intervenes in the IFN-I pathway through epigenetic inhibition of SOCS1 [113]
TCONS_00088786, TCONS_01496394	Kidney tissue	Tubular ischemia and CKD	Affect the expression of genes related to renal fibrosis [87,88]
TUG1	Podocytes	DN	Modulates mitochondrial bioenergetics [59]
	HK-2	AKI	Interacts with Nrf2 [60]
XIST	ECs, TECs	AKI due to renal I/R7 injury	Induces apoptosis and inflammation [80]
	HK-2	Renal calcinosis	Influence the expression of some inflammatory factors [96]
	Kidney tissues and cultured podocytes	MN	Proapoptotic effect through upregulation of Toll-like receptor 4 and downregulation of miR-217 [109,110]
XLOC-032768	HK-2	AKI induced by nephrotoxic agents	Regulates tumor necrosis factor TNF-α [78]

## 4. The Impact of LncRNAs on Stem Cells in Disease

**Figure 2:**
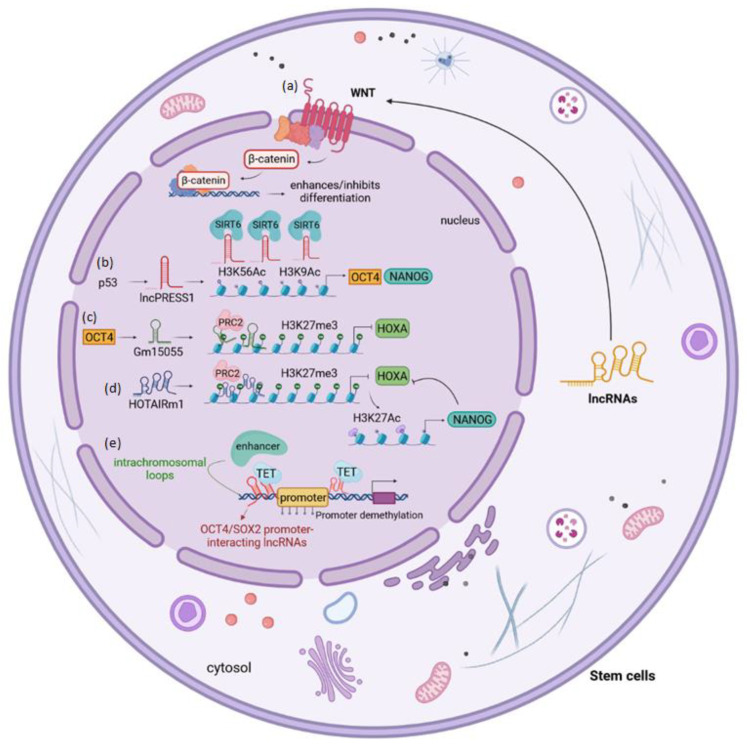
Regulatory mechanisms of lncRNAs in stem cells. (**a**) Several lncRNAs interact in the canonical Wnt signaling cascade and influence the expression of genes by inducing or inhibiting cell differentiation. (**b**) The p53-regulated, long non-coding PRESS1 physically interacts with SIRT6 and inhibits its attachment to chromatin, controlling a gene network that promotes the pluripotency of hESCs (human embryonic stem cells) by maintaining high levels of histone H3K56 and H3K9 acetylation in the promoters of pluripotency genes such as OCT4 and NANOG. (**c**) The lncRNA Gm15055, whose expression is influenced by OCT4, represses HoxA gene expression by recruiting PRC2 to the cluster and maintaining the H3K27me3 modification on HoxA promoters in mESCs (mouse embryonic stem cells); (**d**) The nuclear lncRNA HOTAIRm1 also regulates HoxA expression, leading to trimethylation of histone H3K27 and epigenetic silencing of the gene; furthermore, it promotes the acetylation of H3K27 in the enhancer site of the NANOG gene by upregulating its expression and inhibiting HoxA, which creates a reciprocal regulatory loop that increases the stemness effect. (**e**) Some lncRNAs enhance epigenetic reprogramming by coordinating intrachromosomal looping and by recruiting the TET2 demethylase, promoting DNA demethylation at the OCT4 and SOX2 promoters.

Human adult stem/progenitor cells promote tissue regeneration and play crucial functions in the growth and homeostasis of various tissues. Dynamic interactions between environmental signals, epigenetic factors, and chemicals that control gene expression control stem cell function. LncRNAs are among the highly regarded regulators of stem cell function. In addition to regulating the stemness properties (Figure 2), lncRNAs can also regulate the tissue stem/progenitor cells in response to an insult. Markus Kretz et al., using transcriptome sequencing and tiling arrays, identified transcripts altered during the transition from epidermal progenitors to a differentiated cell population and found that lncRNA ANCR is an important regulator of gene expression required for maintaining the undifferentiated cellular state within the epidermis. In order to influence stem cells’ ability to differentiate into osteoblasts, lncRNAs, such as MALAT1, NEAT1, DANCR, SNHG1, MIR22HG, and LINC00314 may play a role in the development or management of osteoporosis. Most of these lncRNAs’ effects on this process come from their regulation of the PTEN/AKT [114], MAPK [115], and STAT [116] pathways. Cui, Y. et al. instead demonstrated that the lncRNA Neat1 plays an important role in regulating neuronal differentiation, apoptosis, and spinal cord progenitor cell migration by regulating the Wnt/B-catenin signaling pathway activated by miR-124 [117]. The canonical Wnt signaling pathway controls the physiology of stem cells in many other tissues, such as bone, in which the lncRNA AK137033 has been shown in vitro and in vivo to inhibit the osteogenic potential of adipose-derived stem cells in patients with diabetic osteoporosis. The non-coding transcript acts as an epigenetic regulator by modulating the Wnt signaling pathway via DNA methylation in the sFrp2 promoter region [118].

The self-renewal and differentiation capacity of stem cells can be diverted, in some cases, towards a tumorigenic fate. Considering that the regulation of critical signaling pathways, such as polycomb, SOX2, and KLF4 signaling, has been shown to be activated in cancer stem cells and that these involve feedback loops with lncRNAs, it seems likely that lncRNAs could also be involved in promoting the maintenance of cancer stem cells (CSCs) [119,120,121]. For example, HOTAIR was discovered by Gupta et al. as an important lncRNA, whose overexpression is a predictive index of breast cancer metastasis [122] (Figure 2). Future studies on lncRNAs involved in CSC metabolism could lead to considering lncRNAs as clinical biomarkers, indicating which are the best candidates for future therapeutic strategies.

Understanding the potential actions of lncRNAs and their aberrant expression in disease states would allow a wide use in regenerative medicine and pave the way for numerous clinical applications. Current emerging knowledge emphasizes the possibility of considering them as targets for possible therapeutic interventions and pharmacological targets, for example by including complementary sequences within lysosomal vesicles (an approach that has already led to results in pre-clinical and clinical trials) [123] or by transferring them via exosomes to stem cells, a method that could suggest a good strategy to control their differentiation.

## 5. The LncRNAs in Human Adult Renal Stem/Progenitor Cells

As previously described, lncRNAs also play an important role in renal pathogenesis. However, little is known about lncRNAs that are expressed in the healthy kidney and that are involved in renal cell homeostasis and development, and even less is known about lncRNAs involved in human adult renal stem/progenitor cells (ARPC) homeostasis. ARPCs constitute a very promising cell population that has great potential for the development of future treatments for both acute and chronic kidney injury. The ARPCs can be isolated from both tubules and glomeruli; they have many similar morphological and transcriptional characteristics but also important differences [124,125,126,127]. Due to their multipotent differentiation capacity, these cells can give rise to adipocytes, osteogenic cells, and tubular epithelial-like cells. CD133+ CD24+ ARPCs can help with tubular regeneration in mice with acute renal injury brought on by glycerol, and their administration may lessen renal injury and hasten renal repair [124,128,129].

By controlling inflammatory processes and managing the immune response in response to an insult, like exposure to lipopolysaccharides (LPS), ARPCs can aid in the repair of injured kidney tissues by blocking the LPS-induced endothelial to mesenchymal transition through the secretion of the antiseptic molecules CXCL6, SAA4, and BPIFA2 [130,131]. The tARPCs are responsive to insult by chemical agents such as cisplatin, a chemotherapeutic drug that can have nephrotoxic side effects. Following TLR2 activation, renal progenitors secreted inhibin-A and decorin directly as proteins and as mRNA transported in microvesicles, encouraging the proliferation of the remaining cells and preventing the chemotherapic toxicity on the proximal renal tubule epithelial cells (RPTEC) [125].

Additionally, current research has shown that ARPCs can control the immune response by triggering immune system Treg cells and modifying double-negative T-cells (T DN), which are crucial for maintaining the proper balance between immune tolerance and autoimmune disease [132,133].

Renal senescence, which can impact renal progenitors by both inducing renal aging and the inability to repair renal damages, can invalidate all of these regenerative qualities of ARPCs [134]. Several studies have identified long non-coding RNAs as key players in the molecular mechanisms that drive gene regulation, demonstrating that lncRNAs are involved in cellular reprogramming processes [135]. It became very important, therefore, to understand what the lncRNAs role is in the biology of ARPCs.

Very recently, a whole-genome lncRNA expression screening was performed for the first time in ARPCs. About 611 lncRNAs that were differently regulated and capable of discriminating the ARPCs from the RPTECs were discovered. According to the pathway analysis, several lncRNA, exclusively expressed in ARPCs, were shown to be involved in the biological processes regulating the cell cycle. Among differentially modulated lncRNAs, HOTAIR was found to be a crucial component controlling these pathways. By creating HOTAIR knock-out ARPC lines, it was demonstrated how this lncRNA controls ARPC apoptosis and maintains their proliferative and self-renewal abilities.

**Figure 3:**
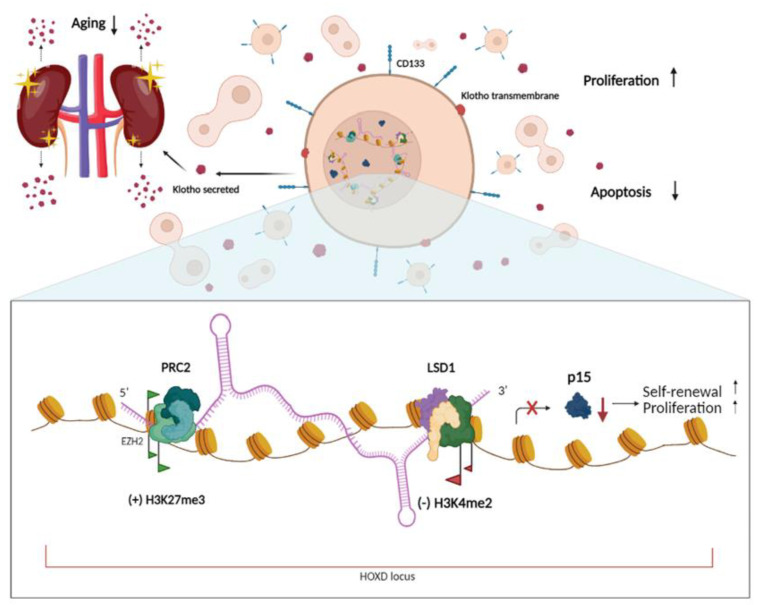
HOTAIR regulates ARPC stemness properties. HOTAIR regulates the proliferation and self-renewal capacity of ARPCs, limits their apoptosis, and regulates CD133 expression, an inverse marker of senescence and functional marker of stemness in ARPCs. HOTAIR also regulates the secretion of α-Klotho, which in turn can influence the surrounding tissues and therefore modulate tissue aging, playing an important role in preventing cells from becoming senescent in the short term. These mechanisms are regulated through p15 epigenetic silencing by HOTAIR. This lncRNA acts as a molecular scaffold to link the PRC2 and LSD1 protein complexes and coordinates the chromatin targeting of these proteins. The complex leads to histone H3K27 trimethylation and H3K4 demethylation in the p15 gene promoter. Trimethylation of H3K27 leads to the silencing of cyclin p15 and helps keep the ARPC cell cycle active, sustaining self-renewal and proliferation. From: Angela Picerno, Francesca Giannuzzi, Claudia Curci, Giuseppe De Palma, Mariagiovanna Di Chiano, Simona Simone, Rossana Franzin, Anna Gallone, Vito Francesco Di Lorenzo, Alessandra Stasi, Giovanni Battista Pertosa, Carlo Sabbà, Loreto Gesualdo, Fabio Sallustio, The Long Non-coding RNA HOTAIR Controls the Selfrenewal, Cell Senescence, and Secretion of Anti-aging Protein Klotho in Human Adult Renal Progenitor Cells, Stem Cells, 2022, Page 11, with permission of Oxford University Press.

Exploiting the CRISPR/CAS9 genome editing method, the HOTAIR fundamental function in maintaining the self-renewal and proliferation of ARPCs has been demonstrated. HOTAIR prevents ARPCs from becoming senescent in the short term by modulating the expression of the CD133 stemness marker. The renal progenitors, thanks to the high expression of HOTAIR, are able to secrete high quantities of α-Klotho, an anti-aging protein capable of influencing the surrounding tissues and therefore modulating renal aging [136] (Figure 3). Emerging data have shown that certain aging-related characteristics in α-Klotho deficient mice may result from stem cell depletion or stem cell differentiation to promote fibrosis; therefore, the dysfunction and depletion of stem cells and progenitor cells contribute to aging [137]. The lncRNA HOTAIR prevents premature depletion of the renal progenitor population in the kidney thanks also to its role in constraining the expression of the cellular inhibitor p15, helping to keep the cell cycle of these progenitor cells active; this action is carried out by methylation of histone H3K27me3 on the promoter of the p15 gene [136]. Through the trimethylation of lysine 27 in histone H3 in the p15 promoter, HOTAIR suppresses the production of the protein p15 in normal ARPCs, favoring growth and self-renewal (Figure 3). Leukemia-causing stem cells have also been identified to use a similar approach [138]. Epidermal stem cells, during the re-epithelialization process of tissues damaged by injury, showed an overexpression of the lncRNA HOTAIR. It plays an important role in the regulation of epidermal stem cells and in the maintenance of in vitro stem cell conditions [139].

HOTAIR is a member of the Hox gene family, a group of homeobox genes that define areas of an embryo’s body plan along the head-to-tail axis of animals. Gene target modulation results from HOTAIR’s recruitment of the PRC2 and LSD1 complexes, which act as a link between them [122,140]. Hundreds of additional genes can be activated or repressed by Hox proteins, a class of transcription factors that can bind to particular enhancer regions on DNA. It is interesting to note that HOTAIR can control HOXc11 and that, together with PAX2, they are crucial for controlling the expression of early genes involved in kidney development [141,142]. This is remarkable since ARPCs are the only adult cells that express the renal embryonic transcription factor PAX2, which is considered a unique identifier of these cells together with CD133 and CD24 markers [124,129].

In addition to being a constitutive marker of renal progenitors, CD133 also limits cellular senescence and functions as a permissive factor for Wnt/beta-catenin signaling, which controls cell proliferation in response to injury and renal tubular repair [143]. As a result, in ARPC, CD133 is a marker inversely correlated with the development of stem cell senescence. In fact, lower Klotho levels, equivalent to those of RPTECs, are produced in ARPCs by the HOTAIR knockout. Secreted Klotho has been found to be essential for the retention of normal proliferation and differentiation in numerous types of stem cells [137]. Proteases break the extracellular domain of this transmembrane protein, which the kidney then releases into the bloodstream. Then, Klotho expression and circulating levels slow down the aging process in the cells of other tissues. It controls a number of aging-related pathways, including insulin signaling, Wnt signaling, and phosphate homeostasis. Additionally, p53/p21, cAMP, protein kinase C, and TGF-β intracellular signaling pathways are all impacted by Klotho [144].

Not much else is known about lncRNAs in human ARPCs. In mouse embryonic kidney cells, among the 17 specific lncRNAs that were identified, Gm29418 had enhancer-like function on a key metanephric mesenchymal (MM) regulatory gene, Six2. This gene regulates the lineage commitment to nephrogenesis and is essential for the self-renewal of MM cells in the developing kidney. It is known that the Hox-Eya-Pax complex and Tcf/Lef1, a part of the Wnt signaling pathway, control Six2 expression [145].

## 6. Conclusions

Numerous studies have demonstrated that lncRNAs express at various levels, are implicated in all phases of renal disorders, and have identified lncRNAs as diagnostic biomarkers. Effectively, compared to miRNAs, lncRNAs have the advantage of being more tissue-specific. LncRNAs may be present as persistent biomarkers in serum and urine. The key to their utility as biomarkers is that they exhibit variable levels of expression in various diseases and are linked to pathways, targets, and events involved in the pathophysiology of kidney disease. However, to the best of our knowledge, few studies have been published in the nephrology field to understand how the lncRNA can mechanistically affect diseases. Instead, we believe that focusing in the future on the study of their mechanisms of action may bring innovation to the therapeutic approach. Various solutions are being studied to target lncRNA, including the delivery of efficient drugs. Aguilar et al. showed that drugs targeting non-coding RNA can be developed by specifically disrupting RNA structure and epigenetic function through small molecules; in particular, they elaborated a screening strategy and identified the compound X1, which binds the prototype lncRNA Xist, suppressing histone H3K27 trimethylation, and blocking the initiation of X-chromosome inactivation [146].

Renal damage progression and LncRNAs are intimately connected, and therapeutic approaches that target these molecules may be helpful to treat renal diseases. MicroRNAs and lncRNAs have a significant role in the regulation of a number of kidney diseases, and the current issue in the field of RNA interference therapy is the creation of novel treatments. Functional miRNAs have been found to be carried by a number of carriers, including exosomes, microvesicles, and high-density lipoproteins, in conjunction with other substances (lipids, proteins, and mRNAs) [147,148]. Specific renal cells can get functional lncRNAs from these naturally occurring nanoparticles. It is also possible to use extrinsic nanotechnology vehicles, such as 13 nm broad gold nanoparticles functionalized with monolayers of RNA molecules modified with alkylthiols. Another technology that is helpful in the field of non-coding RNAs is the CRISPR/cas9 system, which may be used to target specific regions and prevent the long-term expression of RNAs. So, depending on the conditions, it is possible to transfer lncRNAs, miRNAs, or antisense sequences to the kidney, and targeting lncRNAs may be a precise treatment approach for developing kidney illnesses.

## Data Availability

No new data were created or analyzed in this study. Data sharing is not applicable to this article.
